# The molecular mechanism of Zinc acquisition by the neisserial outer-membrane transporter ZnuD

**DOI:** 10.1038/ncomms8996

**Published:** 2015-08-18

**Authors:** Charles Calmettes, Christopher Ing, Carolyn M. Buckwalter, Majida El Bakkouri, Christine Chieh-Lin Lai, Anastassia Pogoutse, Scott D. Gray-Owen, Régis Pomès, Trevor F. Moraes

**Affiliations:** 1 Department of Biochemistry, University of Toronto, 1 King's College Circle, Toronto, Ontario M5S 1A8, Canada; 2 Program in Molecular Structure and Function, Research Institute, The Hospital for Sick Children, Toronto, Ontario M5G 1X8, Canada; 3 Department of Molecular Genetics, University of Toronto, Toronto, Ontario M5S 1A8, Canada; 4 Structural Genomic Consortium, University of Toronto, Toronto, Ontario M5G 1L7, Canada

## Abstract

Invading bacteria from the *Neisseriaceae*, *Acinetobacteriaceae*, *Bordetellaceae* and *Moraxellaceae* families express the conserved outer-membrane zinc transporter zinc-uptake component D (ZnuD) to overcome nutritional restriction imposed by the host organism during infection. Here we demonstrate that ZnuD is required for efficient systemic infections by the causative agent of bacterial meningitis, *Neisseria meningitidis*, in a mouse model. We also combine X-ray crystallography and molecular dynamics simulations to gain insight into the mechanism of zinc recognition and transport across the bacterial outer-membrane by ZnuD. Because ZnuD is also considered a promising vaccine candidate against *N. meningitidis*, we use several ZnuD structural intermediates to map potential antigenic epitopes, and propose a mechanism by which ZnuD can maintain high sequence conservation yet avoid immune recognition by altering the conformation of surface-exposed loops.

## Abstract (web summary)

ZnuD is a conserved zinc transporter expressed in several bacterial pathogens with potential as a target against infection. Here the authors demonstrate a requirement for ZnuD in systemic infection by *N. meningitidis* and define the molecular mechanism of ZnuD-mediated Zinc transport across the bacterial outer membrane.

## Graphical abstract

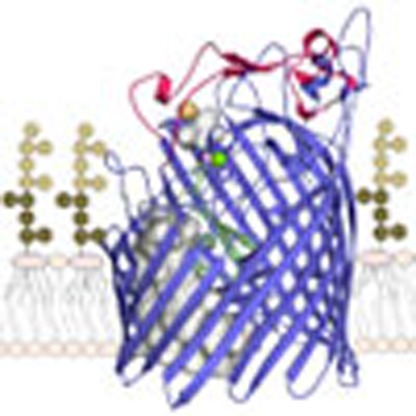

Transition metals, such as iron, zinc and manganese, play essential roles in many biological processes, where they function as enzyme co-factors, regulatory elements or structural factors. To acquire these scarce elements, all organisms express specific transporters and storage proteins. Through a process called ‘nutritional immunity', the mammalian host can sequester these essential metals, restricting availability and preventing colonization by bacterial or fungal pathogens^1^. While the processes related to the host iron sequestration and bacterial iron acquisition are well-described in literature^1^,^2^,^3^, the bacterial zinc uptake systems have emerged as virulence factors in overcoming sequestration from host serum and mucosal surfaces^4^,^5^,^6^. Zinc is the second most abundant transition metal in mammals, while the zinc-binding proteins constitute ∼9% and 5% of the proteome in eukaryotes and prokaryotes, respectively^7^. In mammals, the intracellular zinc concentration is regulated by zinc importers and exporters, metallothioneins and zinc-sensing molecules such as the metal-responsive element-binding transcription factor-1 (ref. 8). The extracellular transport of zinc in plasma is mainly driven by serum albumin, which carries 98% of the exchangeable zinc^9^, leaving only 0.0008% (0.15 nM) of serum zinc in a non-chelated, freely available form^10^. Albumin is the most abundant protein in human serum and is involved in transport of several nutrients such as lipids, haem and metals. Some evidence suggests that albumin may also facilitate zinc uptake from plasma into endothelial cells, potentially via receptor-mediated endocytosis^11^. Zinc levels are further restricted at sites of infection, where neutrophils release calprotectin, a dedicated zinc/manganese-chelating antimicrobial protein that hinders the growth of various pathogenic microorganisms by tightly binding any remaining free zinc ions, thus also contributing to nutritional immunity^12^.

To survive the host environment, pathogens must overcome zinc restriction by expressing high-affinity zinc-transporters such as the zinc-uptake component D (ZnuD), present in various Gram-negative bacteria^12^,^13^. The zinc-uptake protein ZnuD is expressed under zinc-limiting conditions and was originally discovered in *Neisseria meningitidis*^13^, where its expression is under the control of a zinc-sensing transcriptional factor, Zur^14^. ZnuD is an 82 kDa outer-membrane protein predicted to be a member of the TonB-dependent receptor (TbdR) family, requiring the mechanical energy derived from the TonB/ExbB/ExbD inner-membrane complex to couple nutrient uptake to the proton gradient across the inner-membrane^15^. It has been shown that ZnuD expression is induced in human whole blood-cultured *N. meningitidis ex vivo*^16^, while a ZnuD deletion mutant exhibits growth defects in zinc-restricted media^13^. Furthermore ZnuD has also been identified as a crucial resistance factor used by *N. meningitidis* to overcome the innate host defense provided by neutrophils that release DNA/protein neutrophil extracellular traps that immobilize bacterial invaders and hinder the growth of various pathogenic microorganisms^12^.

Considering its essential nature, ZnuD has been targeted as a vaccine candidate against the human pathogen *N. meningitidis*^13^, a leading cause of septicaemia and bacterial meningitis worldwide. *N. meningitidis* asymptomatically colonizes the upper respiratory tract of 10% to 20% of the human population, and occasionally crosses the epithelium to enter the bloodstream to cause invasive disease with a high mortality rate^17^. Of the twelve *N. meningitidis* serogroups classified by their polysaccharide capsule structure, five (A, B, C, W and Y) cause most of the diseases. While capsular polysaccharide-based vaccines are available for four of these pathogenic groups, the development of vaccines against serogroup B (the most common cause of meningococcal disease in developed countries) is more complex due to the resemblance of its capsular polysaccharide to human neural glycoproteins^18^. Thus, protein antigens are being explored as alternative vaccine components, and ZnuD has recently been identified as a promising universal vaccine candidate because it is found in both serogroup B and other meningococcal strains and displays a high level of sequence conservation^19^.

To investigate the role of the vaccine candidate ZnuD during neisserial infection, we initially characterized both the wild-type (WT) and the *znuD* deletion mutant strains of *N. meningitidis* in a mouse infection model. This model provided the first evidence that ZnuD is required to establish systemic infection, promoting blood colonization despite the zinc shortage imposed by the host's nutritional immunity. To understand how ZnuD accesses host-derived zinc, we obtained the first high-resolution crystal structures of this integral membrane protein. Combined with molecular dynamic (MD) simulations, our structural findings suggest that ZnuD acquires free zinc ions using a series of binding sites that move zinc across the outer-membrane through a transient pore that requires TonB activation. Ultimately, our determination of multiple ZnuD structural intermediates provides a critical advance to guide the rational design of ZnuD derivatives that could be used as vaccine antigens to protect against *N. meningitidis* infections.

## Results

### ZnuD is required for neisserial proliferation in blood

**Figure 1:**
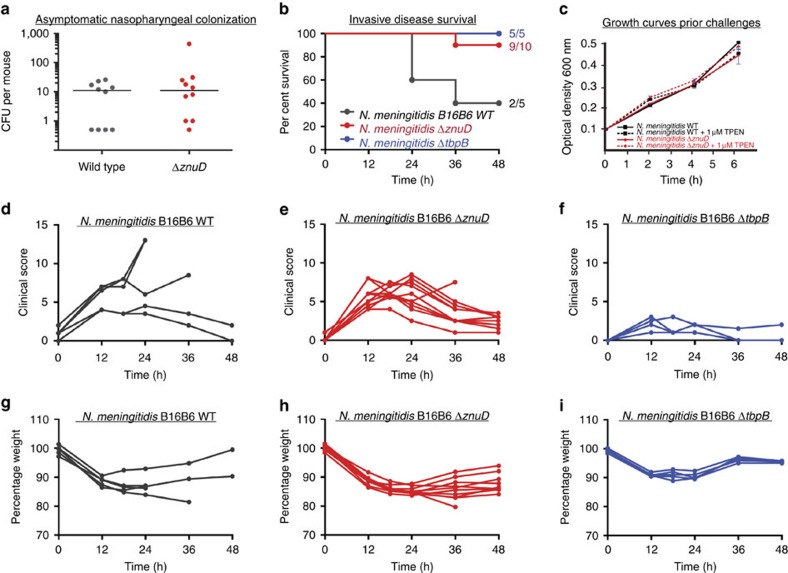
ZnuD is a virulence factor facilitating sepsis infection. (**a**) Mice were infected with ∼1 × 10^7^ colony-forming units (CFU) of *N. meningitidis* strain B16B6 or B16B6 Δ*znuD* via intranasal inoculation. Burden of colonization was assessed after 3 days by recovered CFU from the nasopharyngeal cavity. Each circle represents a single mouse and the bar indicates the group median CFU. No statistically significant difference in colonization burden was identified by a two-tailed Student's *t*-test (*P* value=0.32). (**b**) Survival of infected mice after an intraperitoneal challenge with 10^6^ CFU of *N. meningitidis* B16B6 wild-type, Δ*znuD* and Δ*tbpB* strains. The exogenous human transferrin is the sole source of iron available to *N. meningitidis,* thus the Δ*tbpB* strain is an avirulent positive control. The ratio of survivor mice is indicated for each group. Survival curves of the wild-type and Δ*znuD* groups were compared by log-rank test (*P* value=0.03) to confirm statistically significant difference. (**c**) Growth curves of *N. meningitidis* in RPMI media with and without addition of the zinc chelator TPEN. Bacteria used for the intraperitoneal challenge presented in **b** were initially grown for 4 h in RPMI media supplemented with 1 μM TPEN. (**d**–**f**) Clinical scores for each infected mouse group used in the invasive infection study. (**g**–**i**) Change in body mass for each infected mouse group used in the invasive infection study.

Asymptomatic nasopharyngeal carriage is a necessary first step in neisserial pathogenesis, although the contribution of ZnuD to this process is unknown. In a mouse model of colonization, we observed no difference in the efficiency or burden of colonization when the WT or *znuD*-deleted strains were compared (*P*>0.3) (Fig. 1a). This suggests that *znuD* is not required for bacterial survival at the mucosal surface which is consistent with the assumption that zinc is not limiting in the upper respiratory tract tissues^20^. To better understand the role of ZnuD during neisserial pathogenesis, we then compared the ability of WT or *znuD*-deleted strains from *N. meningitidis* serogroup B strain B16B6 to establish a systemic infection in mice (Fig. 1b). As illustrated in Fig. 1, the *znuD* mutant was less virulent in this model, providing the first evidence that ZnuD confers an advantage for bacterial replication within the host bloodstream (Fig. 1b–i). This is consistent with the predicted function of ZnuD to overcome zinc restriction during mammalian infection^13^. While *znuD* appears to be a critical element for blood colonization, it is relevant to note that *Neisseria* are thought to replicate locally within the sub-epithelial spaces^21^. The elaborate host restriction of *Neisseria* precludes modelling all aspects of infection in mice and it is enticing to consider that ZnuD-mediated acquisition of zinc would facilitate bacterial growth during prolonged (asymptomatic) colonization of humans. Such a scenario would presumably explain the conservation of *znuD* in non-pathogenic *Neisseriaceae* (Supplementary Fig. 1), which exclusively colonize the nasopharyngeal mucosa without causing invasive disease.

### ZnuD is a TbdR promoting zinc uptake

**Figure 2:**
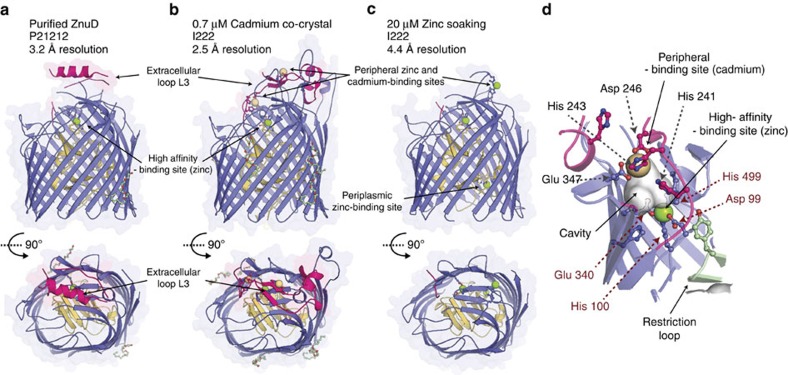
Structural snapshots of ZnuD with distinct zinc occupancies. Side and top structural representations of (**a**) native ZnuD crystals, (**b**) ZnuD co-crystallized with cadmium and (**c**) ZnuD soaked with zinc. The plug domain is coloured gold, the β-barrel domain is represented in blue, the metal-sensing extracellular loop 3 is represented in red, and the co-crystallized detergent molecules are displayed in green and red ball-and-stick representation; the zinc and cadmium ions are illustrated as green and brown spheres, respectively. (**d**) Close-up view of the peripheral and high-affinity binding sites from the cadmium-bound ZnuD crystal structure. ZnuD is represented in blue with the extracellular loop 3 and the restriction loop coloured in red and green, respectively. Cavities are represented as grey surfaces to illustrate the pathway connecting the peripheral-binding site to the buried high-affinity site. Residues contributing to the high-affinity zinc-binding site are labelled with red broken arrows, and residues from the peripheral cadmium-binding site are labelled using grey arrows.

**Figure 3:**
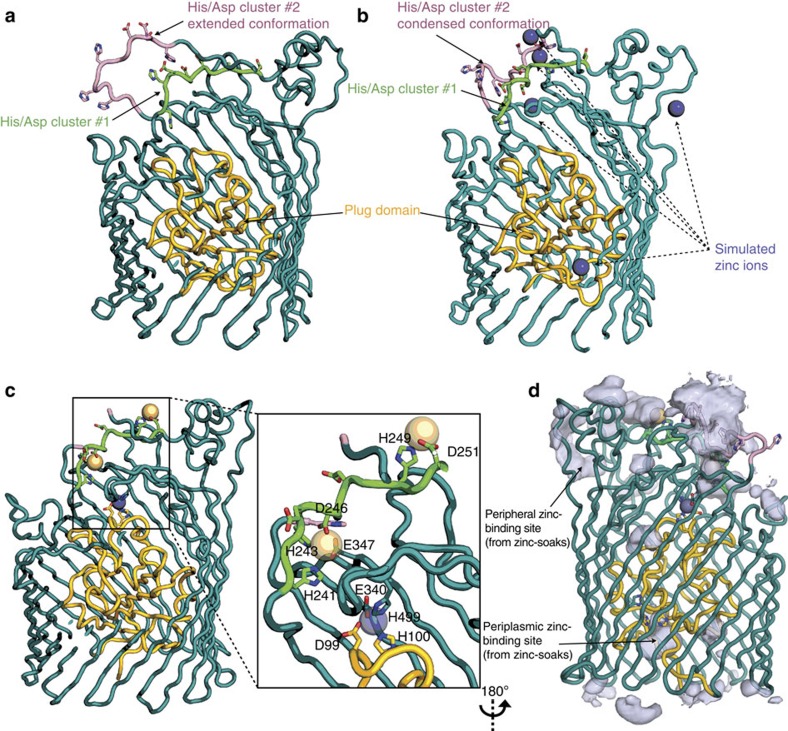
The extracellular loops contribute to zinc sequestration. Snapshots from Supplementary Movie 1 illustrate the putative flexibility of the extracellular loop 3 during the MD simulations of ZnuD in lipid bilayer in the absence (**a**) and presence (**b**) of 100 mM ZnCl_2_. The extracellular loop 3 contains two clusters (coloured pink and green) previously suggested as putative zinc-binding sites^13^ due to the high percentage of histidine, aspartic and glutamic acid residues situated there (drawn in stick representation). Histidines, aspartic and glutamic acids are the high frequency residues coordinating zinc ions within non-structural zinc-binding sites^51^ (structural zinc-binding sites contain His, Asp, Glu and Cys). Cluster #1 (241-HSHEYDDCHAD-251, coloured green) contains two cadmium-binding sites identified within the cadmium co-crystal structure of ZnuD. Cluster #2 (288-HDDDNAHAHTH-298, coloured pink) was shown, through MD simulations, to be a flexible loop that would promote zinc capture by sampling multiple conformational states within the extracellular space. Similarly, the periplasmic zinc-carrier protein, ZnuA, uses a flexible loop with a high ratio of histidine and aspartic acid residues to capture zinc ions^52^. (**c**) Cadmium-bound co-crystal structure of ZnuD in the same orientation as **a**,**b**; the gold and grey spheres indicate the coordinated cadmium and zinc ions, respectively. (**d**) The spatial distribution of zinc was computed using VMD volmap^50^ by averaging post-equilibration frames sampled at an interval of 500 ps on a 1.0-Å^3^ grid, and overlaid onto the cadmium-bound co-crystal structure. Note the co-localizations of the predicted zinc density with the two His/Asp/Glu clusters and the resolved cadmium/zinc binding sites (see Supplementary Movie 1).

To understand the molecular mechanism of zinc-acquisition by ZnuD, we solved several crystal structures of ZnuD in multiple conformations (Fig. 2, Supplementary Fig. 2 and Supplementary Table 1). The structure of the zinc-transporter ZnuD from *N. meningitidis* was solved using single isomorphous replacement with anomalous signal combining native and seleno-derivative ZnuD crystals that revealed a 22-stranded obstructed pore-architecture belonging to the TbdR family^15^. Characteristic of other TbdR family members, ZnuD consists of an amino-terminal plug domain (residues 1 to 147) obstructing the lumen of its own carboxy-terminal pore-forming domain (residues 148 to 734). The transport function of TbdR family receptors requires activation of the plug domain via the inner-membrane protein TonB^15^ (Supplementary Fig. 3), which binds to a conserved Ton-box motif at the amino-terminus of the transporter (residues 10 to 14; disordered peptide unresolved in our three ZnuD structures). ZnuD is the first zinc dedicated TbdR solved to date, as all other TbdR structures that have been solved are related to iron or cobalamin uptake^15^. Other functions of TbdRs that have been reported include the uptake of nickel in *Helicobacter* and carbohydrates in *Caulobacter*^22^. Uniquely, the ZnuD structures illustrate an arrangement in which the extracellular loops act like a fishing net, facilitating zinc acquisition and sequestration. Indeed, equilibrium MD simulations suggest that the conformation of extracellular loop 3 is substrate-dependent. Although this loop collapses and adopts a partially folded conformation in the presence of 100 mM ZnCl_2_, it extends into the extracellular solution when zinc is removed from the simulation (Fig. 3 and Supplementary Movie 1). The high affinity of this TbdR for zinc is exemplified by the observation of a zinc ion bound to ZnuD despite no exogenous zinc addition during the purification. Indeed, all three ZnuD intermediate structures presented in this study contain a zinc ion buried in a high-affinity zinc-binding pocket (Fig. 2 and Supplementary Fig. 4).

### ZnuD shows structural homology with haem receptors

**Figure 4:**
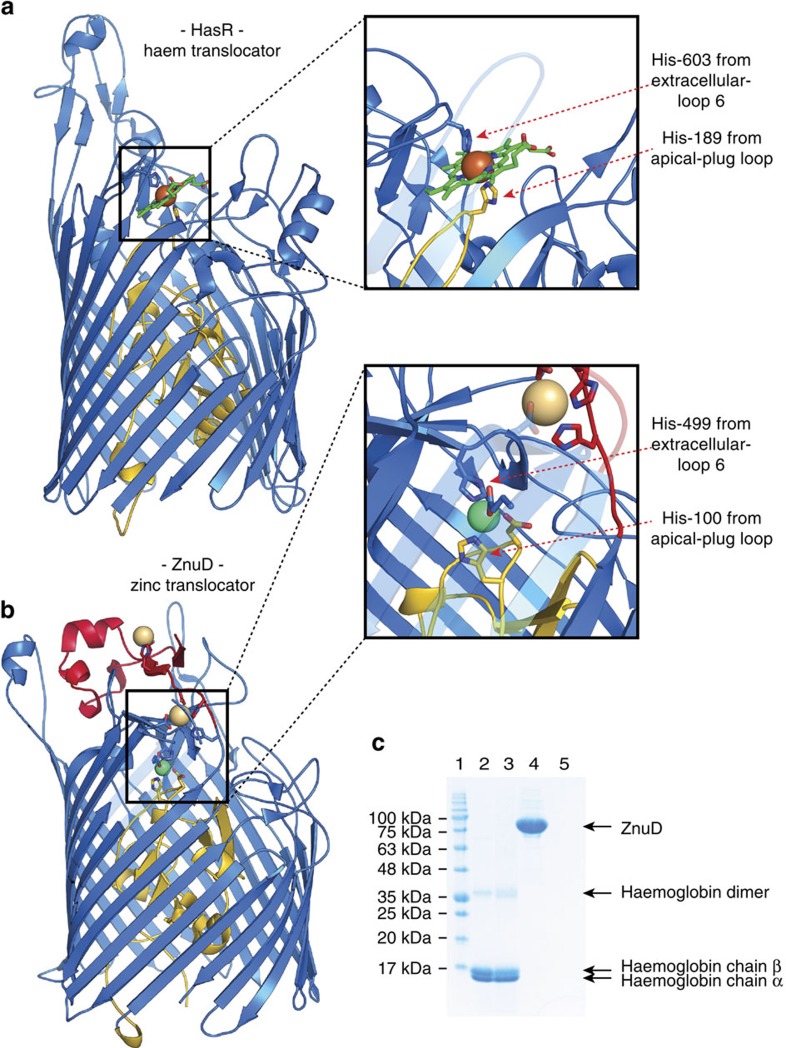
ZnuD is not a haem translocator. Cartoon representation of the TonB-dependent receptors HasR (**a**) (pdb code 3CSL)^2^ and ZnuD (**b**); the boxes highlight an enlarged view of their high-affinity binding sites for haem and zinc, respectively. HasR and ZnuD share a common mechanism to clamp their cognate substrates using two facing histidines from extracellular loop 6 and the plug-apical loop. (**c**) Coomassie-stained SDS–PAGE illustrating the haem-binding profile of ZnuD and haemoglobin using haem-agarose beads. Lane 1 is the molecular weight marker; lanes 2 and 3 are the supernatant and the haem-bead pelleted fractions for the haemoglobin-binding assay (positive control); lanes 4 and 5 are the supernatant and the haem-bead pelleted fractions for the ZnuD-binding experiments.

The metal ion captured within the high-affinity binding site is coordinated by four conserved residues (Asp-99, His-100, Glu-340 and His-499) located in a buried pocket that is sealed by the plug-apical loop and extracellular loop 6 (Fig. 2d). The nature of the endogenous substrate bound to ZnuD in the crystal structures has been further characterized by X-ray absorption spectroscopy to confirm that the bound metal is a zinc ion (Supplementary Fig. 5). This binding site localization overlaps with the location of a haem-binding site identified in the HasR TbdR^2^. Similarly to ZnuD, the HasR receptor docks to its cognate ligand at an interface held by the extracellular loop 6 and the plug-apical loop using two distal histidine residues to clamp the iron metal of the haem molecule (Fig. 4). Although the haem and zinc-binding TbdRs share a similar mechanism to lock their respective ligands, the ZnuD-binding site is not compatible with any potential haem-binding activity. In particular, the 652 Da haem molecule is sterically too large to fit within the narrow zinc-binding pocket deeply buried within the protein. Furthermore, we were unable to co-precipitate ZnuD with haem-agarose beads, illustrating that haem does not bind to purified ZnuD (Fig. 4c). As a consequence, our structures and biomolecular interaction studies do not support ZnuD as a haem translocating protein, which was previously suggested using *E. coli* membrane fractions containing ZnuD receptors^23^. The absence of any metallophore within the binding site suggests that ZnuD is able to sequester and translocate free metal ions from the environment; however, it does not exclude any potential metallophore-binding activity as the neisserial TbdR FrpB/FetA has been reported to acquire its metal ligand either freely or presented through a siderophore^24^,^25^.

### ZnuD adopts multiple conformations on substrate binding

To elucidate the metal transportation mechanism, we attempted to capture ‘snapshots' of ZnuD in multiple translocation binding-states through co-crystallization and by soaking ZnuD with zinc or cadmium ions. The transition metal cadmium, which mimics and competes with zinc-binding sites in proteins^26^, was a critical tool to stabilize and identify two additional binding sites (Fig. 2b). On the other hand, soaking the rigid cadmium-bound conformation of ZnuD with free zinc increased the dynamic flexibility of the protruding loops on interaction with its metal substrate and furthermore identified a potential periplasmic zinc-binding site (Fig. 2c and Supplementary Fig. 5). Our three structures illustrate that ZnuD undergoes drastic surface variations upon substrate binding to peripheral zinc-binding sites. The presence of zinc modulates the dynamic flexibility of the external loops by remodelling an α-helical segment into a pair of β-strand motifs (Fig. 2a,b). Such dramatic rearrangements upon metal-binding has already been reported for other calcium- and zinc-binding proteins^27^,^28^ but is a unique feature of ZnuD within the TbdR family. Together, resolution of three unique receptor intermediates illustrates the ability of ZnuD to capture its substrate using multiple binding sites on flexible loops and, ultimately, drag zinc ions towards a buried high-affinity binding site. This substrate-sequestering step occurs prior to translocation across the membrane through the β-barrel pore that typically requires TonB activation.

### MD simulations of the activated pore

**Figure 5:**
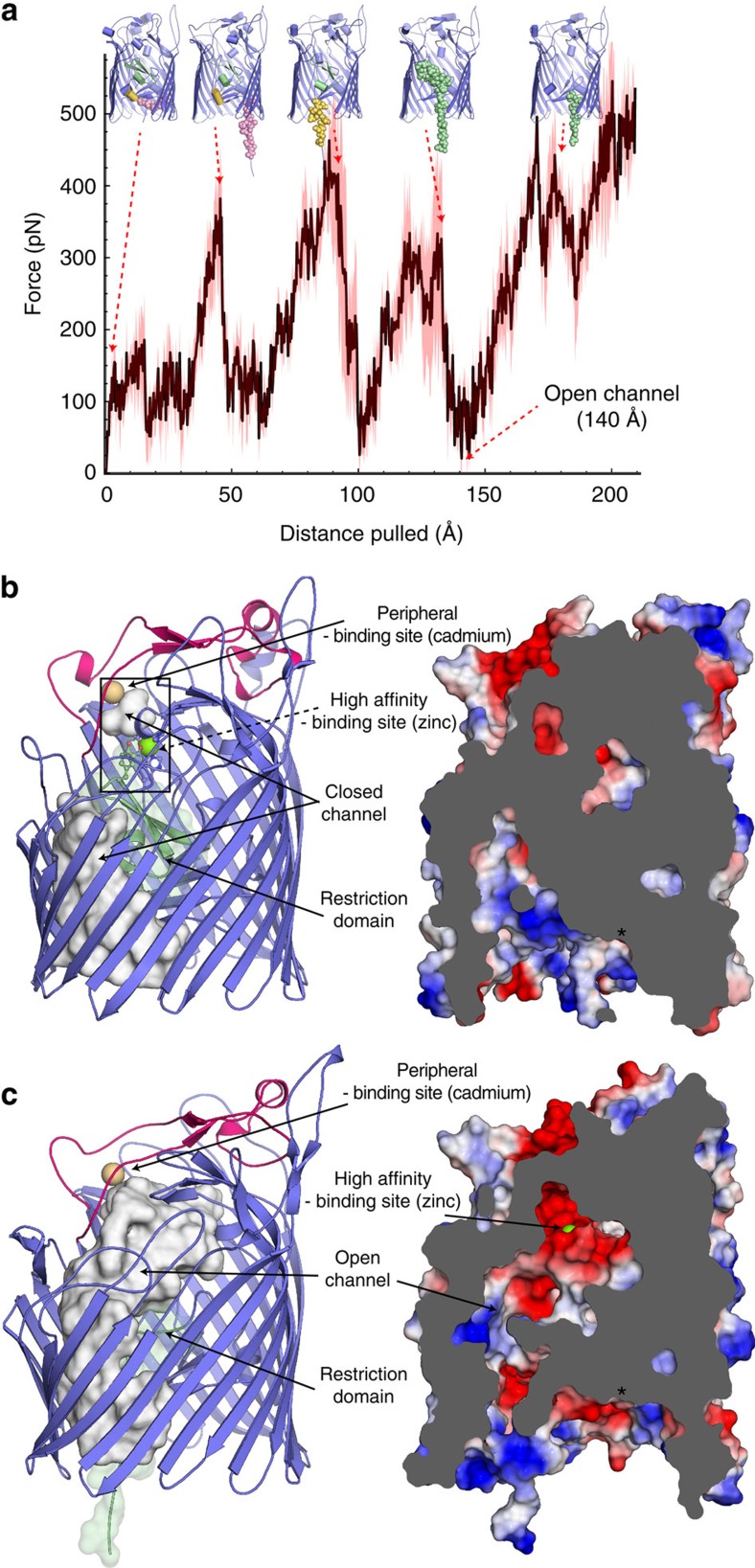
Pathway for zinc translocation across the ZnuD β-barrel. (**a**) Average force profile along the TonB pulling direction from multiple steered MD simulations; the force applied to the amino-terminal residue is shown as a function of the distance its atoms have moved. Translocation intermediates from different time points in the simulation are visualized above the profile. The spikes in the force extension profile correspond to a reproducible sequence of unfolding of three structural motifs, respectively, coloured in pink (residues 15 to 32), yellow (residues 38 to 45) and green (residues 25 to 73). (**b**) Closed (crystal structure) and (**c**) simulated open conformations of ZnuD displayed in cartoon (left panel) and sliced surface (right panel) representations illustrate the transient pore activity upon TonB activation and the displacement of the restriction domain (green). The left panel uses the same colour code as in Fig. 2d with the representation of the cavities along the pore pathway depicted as a grey surface. The right panel depicts the electrostatic potential on the surface of ZnuD; an asterisk indicates the localization of the potential periplasmic zinc-binding site identified in the zinc-soaked structure.

To investigate the pathway for zinc translocation across the ZnuD β-barrel, we used MD to emulate the pulling of the plug domain by the TonB complex^29^. Our simulations suggest that TonB activation induces a partial unfolding of the plug domain (Fig. 5, Supplementary Figs 6 and 7 and Supplementary Movie 2). The spikes in the force extension profile (Fig. 5a) correspond to a reproducible sequence of unfolding of individual secondary structure elements within the plug domain, consistent with the previous investigation of the TbdR protein BtuB^29^. Pulling the plug domain by 140 Å from the N terminus displaces restriction-loop residues 64 to 69 (which obstruct the conduit in the rested conformation) and resulted in a broad water-filled cavity connecting the extracellular to periplasmic sides of the membrane authorizing the formation of a putative ion pathway from the high-affinity binding site to the periplasmic space (Figs 2d and 5b,c).

### Vaccine perspective

**Figure 6:**
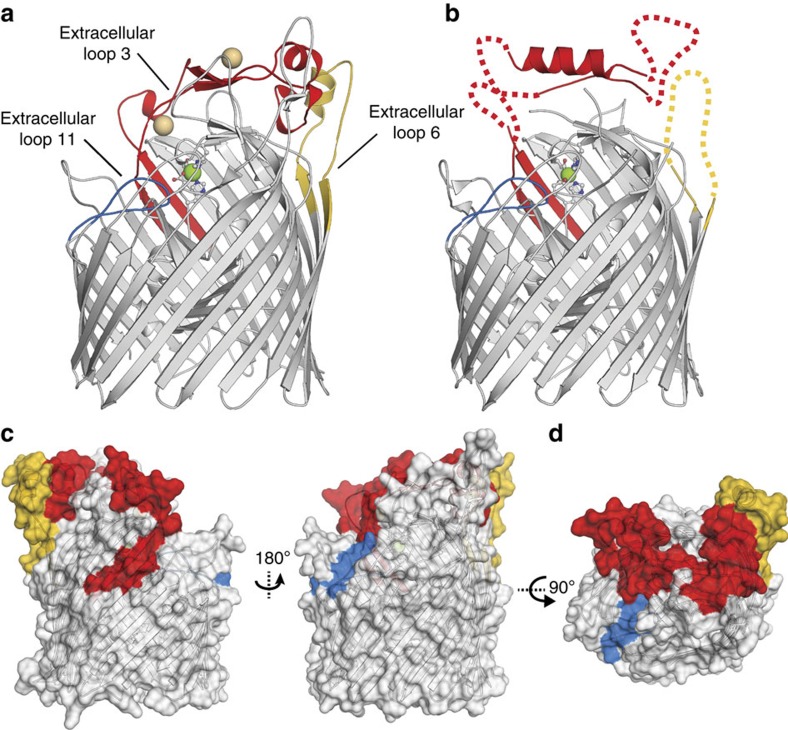
Mapping epitopes on ZnuD. Since immunization with ZnuD elicits bactericidal antibodies against *N. meningitidis*, we have mapped antigenic regions on the surface of the ZnuD structures. The zinc/cadmium-bound (**a**) and the zinc-bound (**b**) ZnuD structures are used to illustrate the most immunogenic peptides previously identified by Hubert *et al.*^19^ from the serum of convalescent patient tested in a ZnuD peptide array experiment. The immunogenic extracellular loops L3, L6 and L11 are coloured in red, gold and blue, respectively. The side (**c**) and top (**d**) view of the cadmium/zinc-bound conformation of ZnuD are shown in surface representations to map the localization of the identified immunogenic epitopes.

Since immunization with ZnuD elicits bactericidal antibodies protecting against *N. meningitidis* serogroups A, B, C and Y, we have mapped and characterized the immunodominant regions on the surface of the ZnuD structure^19^. Specifically, serum from convalescent patients recovering from meningococcal disease was previously used in ZnuD peptide array experiments^19^, revealing that antibodies recognize the ZnuD_233-309_, ZnuD_430-459_ and ZnuD_706-722_ peptides. These peptides mapped onto the structure of ZnuD correspond to the external loops 3, 6 and 11 in our structures (Fig. 6). Interestingly, the most immune-reactive epitopes reported to be targeted by the antibodies from convalescent patient overlaps with the extracellular loop 3, which camouflages the high-affinity zinc-binding pocket. This would presumably prevent the immune system from directly binding and inhibiting this functional site. In addition, on binding its zinc ions on peripheral sites, the immunogenic loop 3 undergoes major conformational changes resulting in a switch between rigid (β-strand pairs; Fig. 2b) and flexible (α-helix; Fig. 2a) conformations. These two dynamic states of the external loop 3 illustrate how this loop adopts various conformations that would ultimately interfere with the surface/sequence availability of this epitope. While most of the outer-membrane proteins from invading pathogens have hypervariable sequences exposed at the cell surface, the conformational heterogeneity observed in ZnuD, a highly conserved and exposed protein (>90% sequence identity based on alignment of 505 *N. meningitidis* strains)^19^, might be a strategy to alter the antigenic surface of this critical TbdR, thus allowing the transporter to avoid recognition and preventing the immune system from mounting a robust response.

## Discussion

The functional and structural data presented here provide the basis for bacterial zinc piracy through the gated pore ZnuD, an important transporter required for the survival and virulence of *N. meningitidis* during invasive disease. We captured three zinc-binding intermediates, which together demonstrate that ZnuD uses a cascade of zinc-binding sites to uptake this scarce element from the host milieu. Initially ZnuD was misannotated as a haem-dedicated transporter^13^,^23^ due to the presence of conserved motifs shared with various haem uptake transporters such as HasR, ShuA and HmbR^23^. This illustrates the difficulties in predicting the substrate spectrum and substrate specificity of the TbdR members. Indeed, our structures confirm the resemblance of ZnuD with other TbdRs involved in haem transport that use a similar mechanism to lock their cognate ligand through two distal histidines from the plug-apical loop and the extracellular loop 6. However, our study shows that purified ZnuD does not associate with haem-agarose beads (Fig. 4), while it captures a free zinc ion within a buried high-affinity binding cavity sized to accommodate a single metal ion interaction (Fig. 2).

In addition, structures of ZnuD are of high interest due to the vaccine potential of this receptor that is able to generate cross-protective bactericidal antibodies against *N. meningitidis*^19^. In particular, the multiple conformations of the extracellular loop 3, the most immunoreactive epitope recognized by the immune system from convalescent patients, raises questions about the purpose of its dynamic sampling of flexible and rigid states upon substrate recognition (Fig. 2). Indeed, the surface remodelling caused by extracellular loop 3 could be a critical element for sequestering zinc ions efficiently, and/or constitute a strategy to keep varying its properties at the bacterial surface, thus preventing ZnuD from being recognized by the immune system. Ultimately, this work allowed us to map the identified immunogenic regions onto the surface of the ZnuD structure and will provide the framework for the design of structure-based ZnuD derivatives to improve its vaccine efficacy.

## Methods

### Bacterial strains and mutants

Isogenic mutants of *N. meningitidis* B16B6 serogroup B were generated by replacing the open reading frames with antibiotic marker genes. The *znuD* mutant strains were generated by transformation of the parental B16B6 WT with the pUCZnud500-Kan plasmid. This plasmids consisted of the 500-bp upstream flanking regions of *znuD* followed by a kanamycin cassette, and the 300-bp downstream flanking region of *znuD,* in pUC19. *N. meningitidis* was transformed by spot transformation^30^. Transformants were selected on BHI plates containing kanamycin (80 μg ml^−1^) and verified by PCR and western blot using mouse immune sera raised against ZnuD. The *tbpB* mutant strain was kindly provided by Dr A.B. Schryvers.

### Mouse colonization study

Intranasal colonization studies were conducted on groups of 10 C57BL/6 mice (mixed-gender evenly distributed over the groups) expressing the human CEACAM1 transgene (bred in-house). Mice were anesthetized with Isofluran (Baxter) and inoculated via intranasal instillation with the animal-passaged *N. meningitidis* strain B16B6 or its *znuD* mutant. To prepare inoculums, bacterial strains for infection were grown overnight on GC agar (BD Biosciences, Canada); the overnight lawn of growth was harvested into 1 ml of PBS containing 1 mM of MgCl_2_ (PBS/Mg) and OD_600_ was measured to adjust the number of bacteria. Cultures were adjusted such that each final 10 μl inoculum contained ∼1 × 10^7^ CFU. Density of colonization dose was confirmed via serial dilution plating on GC agar. Three days after infection, mice were killed by carbon dioxide asphyxiation. Burden of colonization was assessed by swabbing of the nasal passages with a polyester-tipped applicator (Puritan Medical Products) resuspended in 500 μl PBS, and then enumerating colonies after overnight growth on GC agar supplemented with VCNT inhibitor (Becton Dickinson) to prevent growth of nasal flora. Data were analysed by a two-tailed *t*-test using GraphPad Prism version 6.0e; differences were considered statistically significant at *P*<0.05.

### Mouse invasive infection study

Sepsis modelling was performed using groups of 5 or 10 8-week-old male C57BL/6 mice (Charles River) inoculated via intraperitoneal injection with *N. meningitidis* strain B16B6 (*n*=5), B16B6 Δ*tbpB* (*n*=5) or B16B6 Δ*znuD* (*n*=10). To prepare inoculums, bacterial strains for infection were grown overnight on GC agar supplemented with Isovitalex and VCNT inhibitor; resuspensions were grown for 4 h in 10 ml RPMI (Gibco) medium supplemented with 1 μM TPEN at 37 °C with shaking. Cultures were adjusted such that each final 500 μl inoculum contained ∼1 × 10^6^ CFU. Mice received an intraperitoneal injection containing 10 mg human holo-transferrin (Sigma) in sterile PBS (Gibco) immediately prior to challenge. Animals were lightly anaesthetized with isofluorane and the inoculum was administered via intraperitoneal injection. Three hours after bacterial challenge, samples of whole blood were plated on GC agar to confirm the presence of *N. meningitidis* in the bloodstream. Mice were monitored at least every 12 h starting 24 h before infection to 48 h after infection for changes in weight and clinical signs of illness. Mice were scored on a scale of 0–2 based on the severity of each of the following clinical symptoms: weight loss, grooming, posture, appearance of eyes and nose, breathing, dehydration, diarrhoea, unprovoked behaviour and provoked behaviour. Animals reaching endpoint criteria were humanely killed. Survival curves were analysed by Kaplan–Meier method and compared by log-rank test using GraphPad Prism version 6.0e; differences were considered statistically significant at *P*<0.05.

All Animal experiments were conducted in accordance with the Animal Ethics Review Committee of the University of Toronto.

### Protein expression and purification of ZnuD

The region coding the mature protein sequence of ZnuD (NMB0964) from *N. meningitidis* MC58 serogroup B was PCR-amplified from genomic DNA and cloned into a modified pET-26 (Novagen) vector encoding a PelB signal sequence followed by a hexahistidine tag, and a thrombin cleavage site upstream of the insertion site of *znuD*. The resulting recombinant plasmid was transformed into NEB turbo competent *Escherichia coli* and cultured in selective LB-agar medium containing 50 μg ml^−1^ kanamycin. Resulting positive colonies were grown in selective LB, and the recombinant plasmid was purified prior to sequencing.

For large-scale expression, 50 ml of an overnight culture was used to inoculate 2 l of Terrific Broth media supplemented with 50 μg ml^−1^ kanamycin. Expression of the recombinant ZnuD was induced by addition of a final concentration of 0.5 mM isopropyl-1-thio-Beta-D-galactopyranoside when the cell density reached an OD_600_ between 2.0 to 3.0 a.u. After 18 h incubation at 30 °C, cells were harvested by centrifugation and resuspended in cold extraction buffer containing 20 mM HEPES pH 8.0, 250 mM NaCl, 1 mM phenylmethylsulfonyl fluoride and 2% (w/v) Elugent (Calbiochem). The whole cell lysate was incubated for 12 h at 4 °C under gentle agitation, and bacterial debris was removed by centrifugation at 30,000*g*. The detergent-solubilized extract was incubated with 4 ml Ni-NTA resin (Thermo Scientific), which was ultimately collected in a chromatography column after 6 h. The resin was washed with 30 column volumes of buffer A (20 mM Hepes pH 8.0, 150 mM NaCl and 0.6% C_8_E_4_) containing 40 mM imidazole, then the bound His-tagged ZnuD was eluted with buffer A containing 200 mM imidazole.

The eluted protein was concentrated to 10 mg ml^−1^ using a 100 kDa cutoff Amicon Ultra centrifugal filter (Millipore) and loaded onto a Superdex200 size-exclusion column (GE Healthcare). ZnuD ran as a single elution peak in 20 mM HEPES pH 8.0, 150 mM NaCl and 0.6% C_8_E_4_, which was used as the mobile phase buffer. Purity was verified using SDS–PAGE and recombinant ZnuD was concentrated to 20 mg ml^−1^ and kept at 4 °C for storage.

Selenomethionine (Se-Met)-labelled ZnuD was expressed using the methionine auxotrophic *E. coli* BL21 (DE3) B834 strain cultured in auto-inducing P-5052 minimal media supplemented with Se-Met^31^, and was purified following the same protocol as for native protein.

### ZnuD crystallization

Native and Se-Met ZnuD crystals were optimized at 20 °C using hanging drop vapour diffusion. A first crystal form in space group P2_1_2_1_2 was initially grown at a protein concentration of 20 mg ml^−1^ in a mother liquor composed of 0.9 M MgSO_4_, 100 mM Bis-tris-propane pH 7.0 and a 1:1 proteinprecipitant ratio. A second I222 crystal form was obtained using cadmium co-crystallization of 5 mg ml^−1^ ZnuD mixed with 0.7 mM CdCl_2_, 0.35 M MgSO_4_, 100 mM sodium cacodylate pH 6.7 and 2% ethylene glycol (w/v), at a 2:1 protein:precipitant ratio. As ZnuD did not co-crystallize with zinc, we soaked several I222 crystals with 10–50 mM ZnSO_4_ for 1–60 h. All crystals were cryo-protected in mother liquor supplemented with 20% glycerol (w/v) and 5–20 mg ml^−1^ ZnuD, and flash-frozen in liquid nitrogen.

### Structure determination

As molecular replacement with a combination of previously solved TbdRs failed to yield phases for ZnuD, we expressed a Se-Met-labelled ZnuD protein to collect experimental phase information on the I222 crystal form. Native and Se-Met diffraction data were collected at 12.66 KeV (selenium-edge) and 9.92 KeV (zinc-edge) and processed with X-ray diffraction studies (XDS). The I222 crystal form was solved by the single isomorphous replacement and anomalous signal (SIRAS) method using ShelX, which located 10 heavy atom sites attributed to 7 selenium, 1 zinc and 2 cadmium ions. The SIRAS phasing was performed using a 2.8 Å native data set combined with an isomorphic 3.5 Å seleno-derivative data set containing useful anomalous signal to 4.5 Å. Data collection and refinement statistics are summarized in Table 1. The initial map was improved by density modification (72% solvent) using Phenix Autosol^32^; a poly-alanine model was manually built and a final model was generated following several rounds of model building and refinement using Coot^33^ and Phenix^32^. The refinement led to an *R*_work_ of 21% and an *R*_free_ of 24% using a 2.4 Å data set. The soaking of the I222 crystals with zinc induces major structural changes within ZnuD that contribute to a rearrangement of the crystal packing. The zinc soaked crystals usually diffracted <6 Å resolution independently of incubation time (30 min to 3 days) or zinc concentration (10–50 mM). Nevertheless, a successful zinc soaked data set was collected at the zinc and selenium edge from two rare crystals diffracting to 4.4 and 4.9 Å, and solved by molecular replacement using Phaser^32^. The final round of refinement using experimental phases and tight geometric constraints led to an *R*_work_ of 31% and an *R*_free_ of 37%. The ZnuD crystals grown in space group P2_1_2_1_2, were solved at 3.2 Å resolution by molecular replacement using the cadmium-bound ZnuD structure as a search model. The final model was obtained after several building and refinement cycles using Phenix and Coot, and yielded a final *R*_work_ of 27% and *R*_free_ of 30%.

The numbering of residues initiates with the first mature amino acid (His-1) remaining after signal peptide cleavage.

### Haem-agarose-binding assay

The haem-binding assay was performed by incubating 40 μl of haem-agarose beads (Sigma-Aldrich) with 1 ml of purified ZnuD or haemoglobin (positive control) at a concentration of 2 mg ml^−1^ in binding buffer (20 mM HEPES, pH 7.5, 200 mM NaCl and 0.5% C_8_E_4_ detergent) for 15 min at 4 °C. The resin was collected by centrifugation at 9,000*g* for 5 min then washed in 1 ml of binding buffer for 5 min under gentle agitation. The resin was washed four times then resuspended in 30 μl of binding buffer. The supernatant fraction containing the unbound proteins, and the final haem-agarose bead fraction containing the haem-bound proteins were then analysed by SDS–PAGE (sample denatured in 2 × Laemmli buffer for 2 min at 60 °C). The polyacrylamide gel was ultimately stained with Coomassie brillant blue R250 to visualize proteins.

### Simulation set-up

Models of ZnuD were generated with the Cd-soaked crystal structure (2.47 Å resolution, PDB entry 4RDR). The missing loop segment (sequence 287-FHDDDNAHAHT-297) was constructed using the best-scoring conformation out of 15 models generated using the ‘DOPE loop model' algorithm in MODELLER 9.11 (ref. 34). The software Lambada^35^ was used to determine the hydrophobic surface of the protein and orient it within a pre-equilibrated lipid patch of 512 lipids. The initial coordinates of the POPC (1-palmitoyl-2-oleoyl-sn-glycero-3-phosphocholine) bilayer were obtained from Stockholm Lipids (128 POPC, 303 K, http://mmkluster.fos.su.se/slipids/). The protein was membrane embedded using the Inflategro2 protocol with 50 alternating deflating and energy minimization steps where protein overlapping lipids were removed^35^. Eighty-seven crystallographic water molecules were kept within ZnuD. In simulations containing ZnCl_2_, three crystallographic Zn ions were kept at the H249/D251-, H241/H243/D246/E347- and D90/H100/E340/H499-binding sites. Both the N- and C-terminal ends of the protein were modelled as neutral moieties and all residues had standard titration states predicted for amino acids at pH 7. The protein and bilayer were solvated in an orthorhombic simulation cell of dimensions 12.6 × 13.2 × 9.6 nm^3^. In simulations containing ZnCl_2_, we used the ‘genion' application to insert ∼100 mM ZnCl_2_ based on the approximate volume of water in the simulation cell (53 Zn^2+^, 110 Cl^−^)^36^. In simulations without ZnCl_2_, four Cl^−^ counterions were included to neutralize the net charge of the protein. Final simulation cells included the ZnuD protein, a POPC bilayer of 445 lipid molecules and 32,544 water molecules (∼168,630 atoms). The protein, lipids and ions were modelled with the CHARMM36 all-atom force field^37^,^38^ and the TIP3P model was used for water molecules^39^. The non-polarizable Zn^2+^ atom in the CHARMM force field reproduces the ion–water radial distribution function obtained from quantum mechanical calculations, but overestimates ion–water coordination energies^40^. As such, we expect similar strong binding to oxygen ligands of ZnuD side chains, preventing brute-force sampling of Zn^2+^ translocation on the timescale of hundreds of nanoseconds.

### MD simulation details

All simulations were performed with GROMACS 4.6.5 (ref. 36) with a time step of 2 fs. Lennard–Jones interactions were evaluated using a group-based cutoff for separation distances <1.2 nm. Coulomb interactions were calculated using the smooth particle-mesh Ewald method^41^,^42^ with a real-space cutoff of 1.2 nm and a Fourier grid spacing of 0.16 nm. The non-bonded pair-list was updated every 10 fs. Simulation in the isothermal-isobaric ensemble (NPT) ensemble was achieved by semi-isotropic coupling to a Parrinello–Rahman barostat^43^,^44^ at 1 bar with coupling constants of 2 ps; temperature coupling was included using the Nosé–Hoover thermostat^45^,^46^ at 300 K with a 0.5 ps coupling constant for protein, bilayer and aqueous solution coupling groups. All covalent bonds were constrained with SETTLE^47^ and P-LINCS^48^ for water and other molecules, respectively.

ZnuD was prepared for production sampling using a gradual equilibration process. Steepest-descent energy minimization was performed to ensure forces <1,000 kJ mol^−1^ nm^−1^. Step-wise thermalization in the NPT ensemble was used to increase temperature linearly from 0 to 300 K over 0.5 ns with protein heavy atoms restrained and the lipid phosphorus atoms restrained to move only in the *x*–*y* plane. All positional restraints were held with a force constant of 1,000 kJ mol^−1^ nm^−2^. Annealing was followed by 0.5 ns of simulation in the canonical ensemble (constant N, constant V, and constant T) and 1 ns of NPT simulation, both of which were conducted with protein heavy atoms restrained and without lipid restraints. Two subsequent 1.0 ns blocks of simulations were performed in the NPT ensemble, the first with backbone-only restraints and the second with Cα-only restraints.

Production simulations consisted of multiple repeats with different random initial velocities for 100 mM ZnCl_2_ simulations (five repeats, 500 ns) and 0 mM ZnCl_2_ simulations (five repeats, 250 ns), summarized in the Supplementary Table 1. Fifty-three Zn^2+^ and 110 Cl^−^ ions were then added to the final frames of each of the five simulations at 0 mM ZnCl_2_, and 250 ns of simulation was performed in this new system. Each of these five new simulations was repeated for a total of 10 simulations. Total times of 1.25 and 5 ms of simulation of ZnuD in a hydrated bilayer were performed in the absence and presence of ZnCl_2_, respectively.

### Steered MD system preparation

Four additional simulations were extended from the last frame of four of the 100 mM ZnCl_2_ trajectories, without the presence of a bilayer (four repeats, 500 ns). These systems were prepared with only ZnuD and all water molecules and ions within 5 Å of the protein. This system was resolvated and ionized in a rhombic dodecahedron box with a minimum protein-box distance of 1.0 nm (volume 1,157 nm^3^). Each of these systems contained the ZnuD protein, ∼34,260 water molecules, 53 Zn^2+^ and 110 Cl^−^ ions. To eliminate β-barrel fluctuations in the absence of a bilayer, simulations included 418.5 kJ mol^−1^ nm^−2^ harmonic restraint potentials on the backbone atoms of ZnuD β-barrel (defined as residues 148–188, 207–237, 303–364, 371–386, 397–433, 457–486, 515–544, 575–607, 639–661, 678–702 and 725–734). Two thousand steps of steepest-descent energy minimization were performed prior to production simulations. All simulation forcefields and parameters were identical to the lipid-embedded system with the exception that isotropic pressure coupling was used.

From the final frame of the four aqueous barrel-restrained simulations, we performed a pulling experiment to unfold the plug domain inside ZnuD using the GROMACS pull code^36^. This procedure was done in accordance with the protocol described by Gumbart *et al.*^29^, wherein the terminus of the plug domain was repeatedly pulled until it was fully extended and then truncated. Pulling was achieved with a distance-based restraint in the *z* dimension (along the primary axis of the protein) between the centre of mass of the β-barrel Cα atoms and the N-terminal nitrogen atom. The distance between these groups increased at a rate of 0.5 Å ns^−1^ and was enforced with a force constant of 418.4 kJ mol^−1^ nm^−2^. Due to the limited simulation cell size in the *z* dimension, pulling was done in four 100 ns blocks, whereby the N-terminus was extended ∼50 Å per block before a stretch of ∼15 residues were truncated (Supplementary Table 1). In each pulling block, depending on the amount of protein that was truncated, Cl^−^ counterions were added or removed to maintain a net zero charge.

### MD analysis details

All trajectories were aligned with respect to the β-barrel Cα atoms of the initial membrane-embedded structure before system equilibration (in the calculation of root-mean-square (r.m.s.) deviation, r.m.s. fluctuations and spatial distributions). To improve the statistics of ensemble averages computed over each respective system, the initial 50 ns of data were removed from each of the 100 mM ZnCl_2_ and 0 mM ZnCl_2_ trajectories. No data was removed from the beginning of re-added 100 mM ZnCl_2_ trajectories or any of the aqueous ZnuD trajectories. Error bars on all simulation data were computed as the standard error of the mean over all simulation repeats. R.m.s. calculations (Supplementary Figs 6 and 7) were performed using MD analysis^49^. Spatial distributions of zinc and water oxygen atoms were computed using VMD volmap^50^ by averaging post-equilibration frames sampled at an interval of 500 ps on a 1.0 Å^3^ grid. Water density along the proposed zinc permeation pathway was computed by extracting oxygen atom positions within the β-barrel and within 10 Å of selected residues (169, 181–182, 203, 254–256, 528 and 750).

## Additional information

**How to cite this article:** Calmettes, C. *et al.* The molecular mechanism of Zinc acquisition by the neisserial outer-membrane transporter ZnuD. *Nat. Commun.* 6:7996 doi: 10.1038/ncomms8996 (2015).

## Supplementary Material

Supplementary Figures and Supplementary Table Supplementary Figures 1-7 and Supplementary Table 1

Supplementary Movie 1 The extracellular loops contribute to zinc sequestration. The movie S1 illustrates the putative flexibility of the extracellular loop 3 during the MD simulations of ZnuD in lipid bilayer in absence and presence of 100 mM of ZnCl2. The plug and barrel domains are coloured in yellow and blue respectively, the extracellular loop 3 is drawn in red, and the simulated zinc ions are represented as green spheres. Movie S2: Unfolding of the plug domain. The supplementary movie 2 illustrates the plug domain unfolding at a pulling speed of 0.5 Å/ns as described in the method section. The plug and barrel domains are coloured in yellow and blue respectively, the extracellular loop 3 is drawn in red, and zinc ions are represented as green spheres.

Supplementary Movie 2 Unfolding of the plug domain. The supplementary movie 2 illustrates the plug domain unfolding at a pulling speed of 0.5 Å/ns as described in the method section. The plug and barrel domains are coloured in yellow and blue respectively, the extracellular loop 3 is drawn in red, and zinc ions are represented as green spheres.

## Figures and Tables

**Table 1:** Refinement statistics for the three ZnuD-crystal structure intermediates solved in this study.

	**ZnuD**	**Cadmium (0.7 μM) co-crystallized ZnuD**	**Zinc-soaked (20 μM) ZnuD**
PDB code	4RDT	4RDR	4RVW
Phasing method	MR	SIRAS	MR
Data collection*			
Space group	P2_1_2_1_2	I222	I222
Cell dimensions			
*a*, *b*, *c* (Å)	101.3, 156.2, 158.0	101.0, 155.8, 159.6	101.5, 149.6, 157.6
*α*, *β*, *γ* (°)	90, 108.5, 90	90, 90, 90	90, 90, 90
Wavelength (Å)	0.9793	0.9793	1.269
Resolution (Å)	49.9–3.20 (3.31–3.20)	40.0–2.47 (2.56–2.47)	49.6–4.47 (4.64–4.47)
*I*/*σI*	14.1 (3.6)	13.4 (2.4)	9.0 (2.9)
Completeness (%)	99.9 (99.8)	99.9 (99.5)	99.8 (99.7)
Redundancy	8.1 (8.0)	15.0 (15.2)	12.9 (13.4)
*R*_sym_	0.12 (0.57)	0.14 (1.75)	0.25 (1.76)
CC_1/2_	0.99 (0.93)	0.99 (0.83)	0.99 (0.99)
			
Refinement*			
Resolution (Å)	49.9–3.20	40.0–2.47	49.6–4.4
No. of reflections	42,108 (4,130)	45,376 (4,479)	7,512 (737)
*R*_work_/*R*_free_	0.27/0.30	0.21/0.24	0.31/0.37
No. of atoms	10,159	5,985	4,839
Protein	10,076	5,633	4,831
Ligands	72	257	8
Waters	11	95	0
B-factors			
Protein	123.4	90.5	253
Ligands	116.8	115.9	290
Waters	66.1	91.3	
R.m.s deviations			
Bond lengths (Å)	0.008	0.008	0.004
Bond angles (°)	1.57	1.20	0.74
Ramachandran			
Favored (%)	91.6	93.0	92.3
Outlier (%)	0.3	0.1	0.3

MR, molecular replacement; SIRAS, single isomorphous replacement with anomalous signal; ZnuD, zinc-uptake component D

^*^(Highest resolution shell is shown in parenthesis).
