# Supplementary material for: The molecular mechanism of Zinc acquisition by the neisserial outer-membrane transporter ZnuD
Source: Nat Commun. 2015 Aug 18;6:7996. doi: 10.1038/ncomms8996 (PMC4557270; doi:10.1038/ncomms8996)
Supplement: Supplementary Figures and Supplementary Table — Supplementary Figures 1-7 and Supplementary Table 1 [file ncomms8996-s1.pdf]

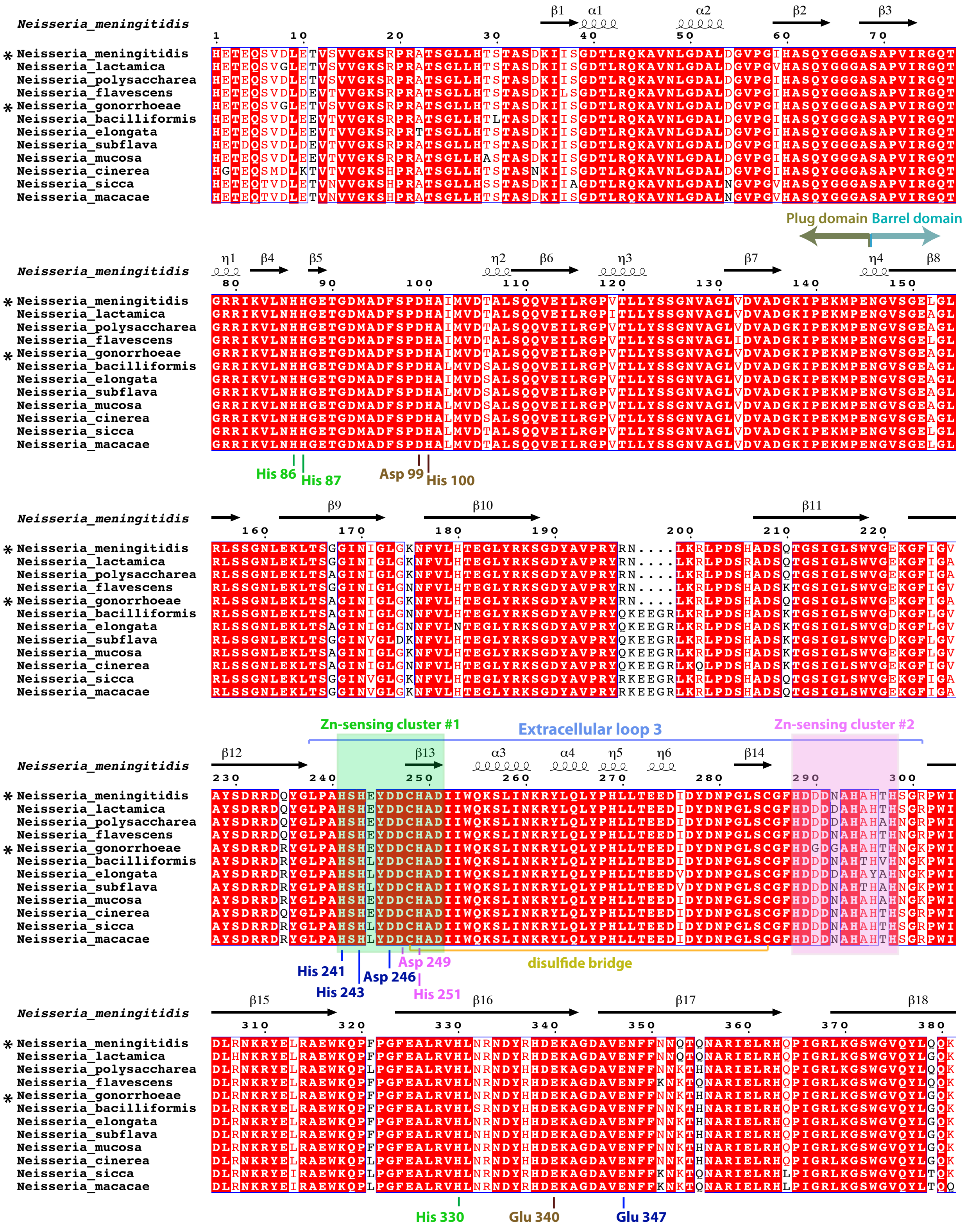

Neisseria_meningitidis
Neisseria_lactamica
Neisseria_polysaccharea
Neisseria_flavescens
Neisseria_gonorrhoeae
Neisseria_bacilliformis
Neisseria_elongata
Neisseria_subflava
Neisseria_mucosa
Neisseria_cinerea
Neisseria_sicca
Neisseria_macacae
Plug domain
Barrel domain
His 86
His 87
Asp 99
His 100
Zn-sensing cluster #1
Extracellular loop 3
Zn-sensing cluster #2
His 241
His 243
Asp 246
Asp 249
His 251
disulfide bridge
His 330
Glu 340
Glu 347

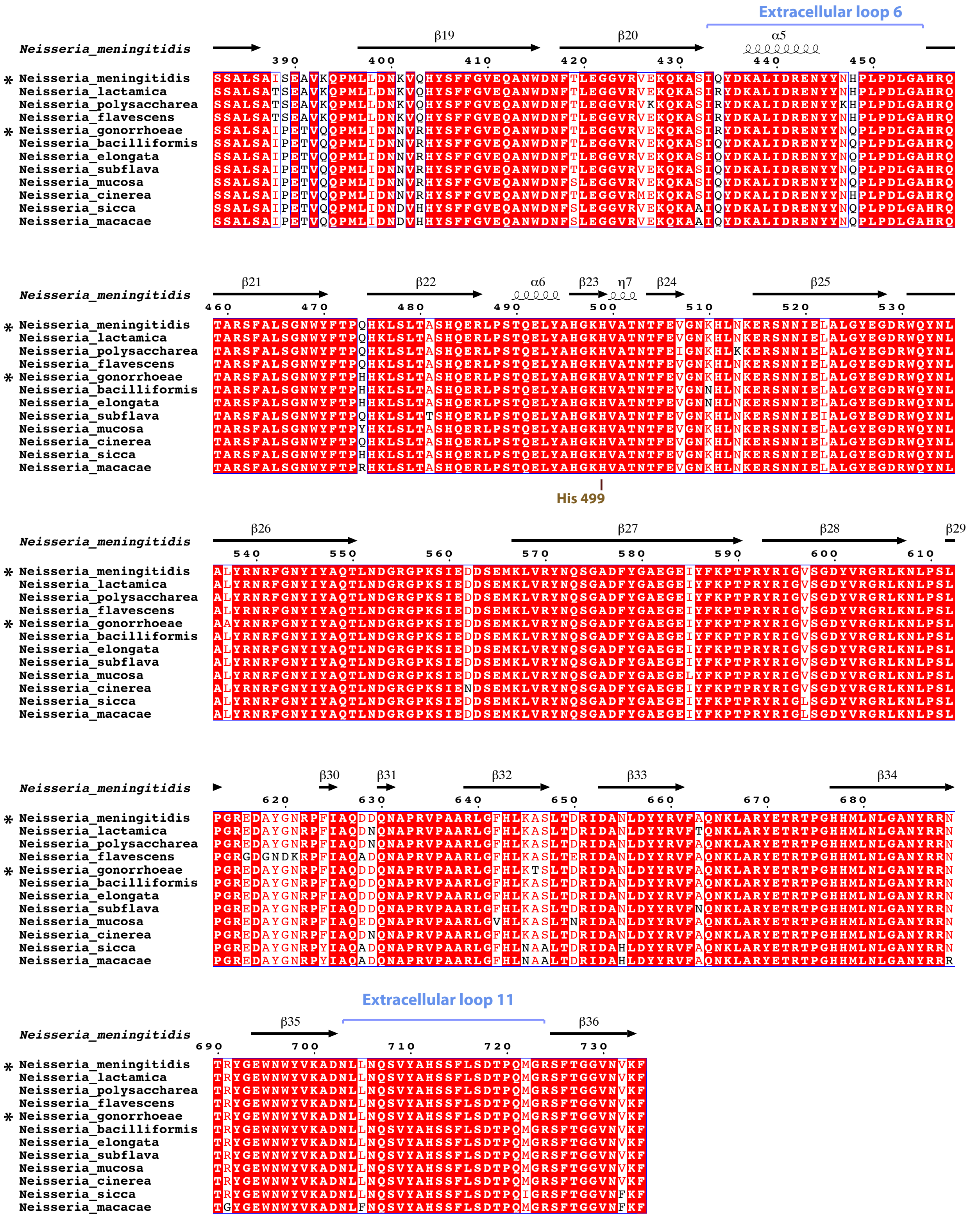

Supplementary figure 1: Sequence alignment of ZnuD from various representative species within the Neisseriaceae family. All sequences start from the first mature

residue remaining after the signal peptidase cleavage; identical and conserved residues are highlighted and boxed in red. The important residues that contribute to the high affinity Zn-binding site and the periplasmic Zn-binding site are indicated in brown and green respectively, while the residues from the two Cd-binding sites are labeled in blue and pink. The secondary structure elements observed from the Cd-bound crystal structure of *N. meningitidis* are shown above the sequences. An asterisk indicates the pathogenic neisseriaceae species.

A

B

C

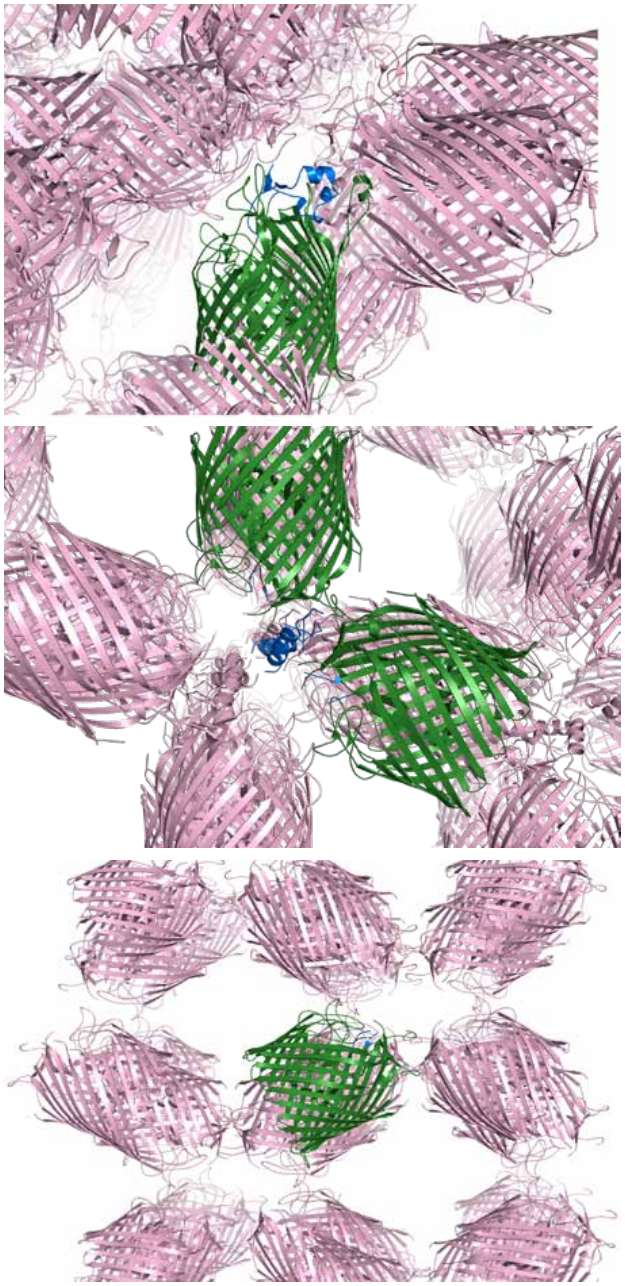

Supplementary figure 2: Crystal packing of the three ZnuD structural intermediates. Illustration of the crystal packing of the Cd co-crystallized (**A**), native purified (**B**), and the Zn-soaked ZnuD structures (**C**). The protein content from the asymmetric unit is drawn in green with the extracellular loop 3 displayed in blue. We notice that the extracellular loop 3 is not constrained by crystal contacts to artificially adopt either a rigid (**A**) or a flexible (**B** and **C**) conformation.

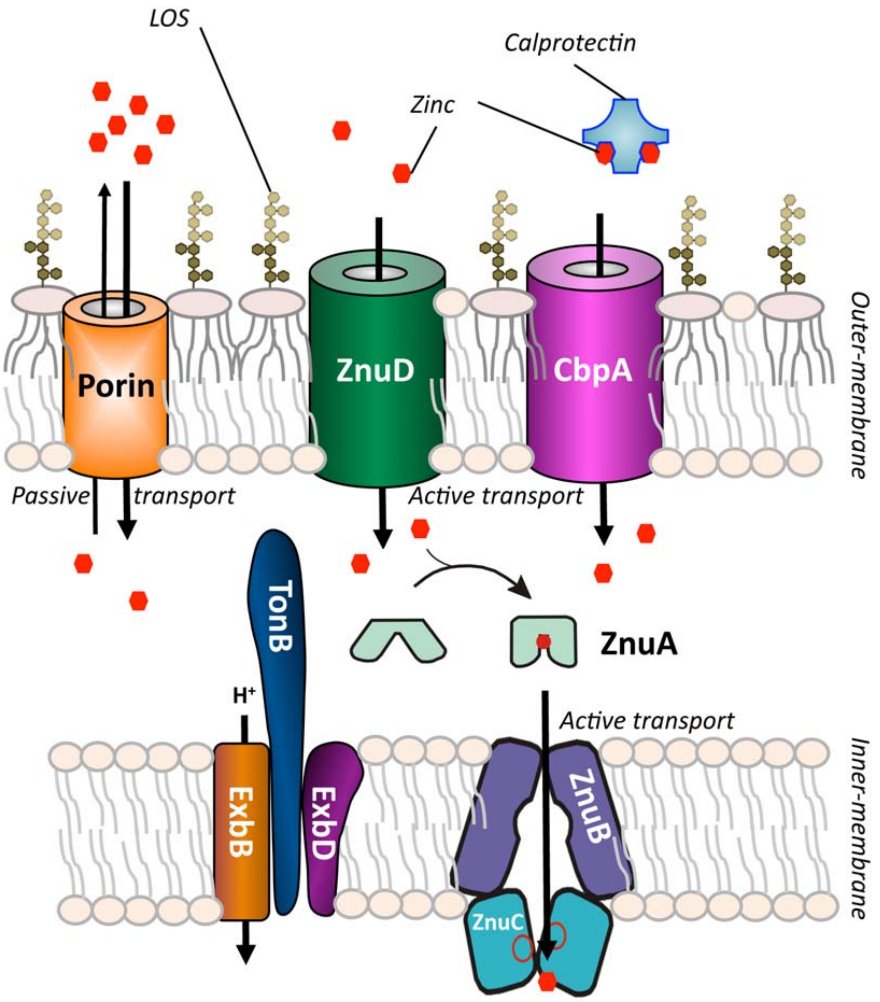

Supplementary figure 3: Schematic of the *Neisserial* high affinity Zn-transporters. In Zn-restricted conditions, *Neisseria meningitidis* expresses high-affinity, Zn-specific transporters including the calprotectin-binding protein A (CbpA)[6], and the four zinc uptake components ZnuABCD[1,13]. CbpA and ZnuD are two TonB-dependent receptors responsible for the active transport of zinc across the outer-membrane, acquiring zinc ions from the human protein calprotectin (antimicrobial Mn/Zn-chelating peptide secreted by epithelial cells and macrophages) and the pool of labile/free zinc, respectively. While in the periplasm, zinc ions are captured by the periplasmic Zn-binding protein ZnuA, which carries the metal substrate to the ABC transporter ZnuBC to translocate the zinc ions into the cytoplasm in an ATP-dependent manner. The *cbpA* and *znuABCD* genes are all under the regulation of the Zn-sensing transcriptional factor Zur, and are over-expressed in zinc deficient conditions[14].

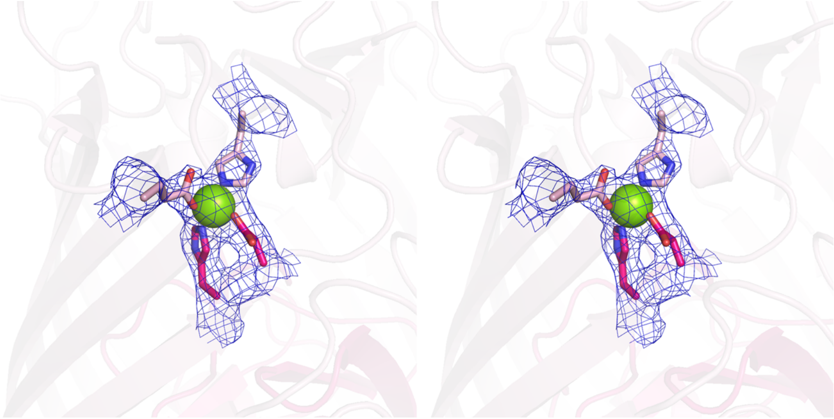

Supplementary figure 4: Electron density map for the high affinity Zn-binding site. The electron density of the high-affinity Zn-binding site from the cadmium co-crystallized structure of ZnuD (pdb code 4RDR). The 2Fo-Fc map is visualized as a stereo image contoured at 1.2 sigma.

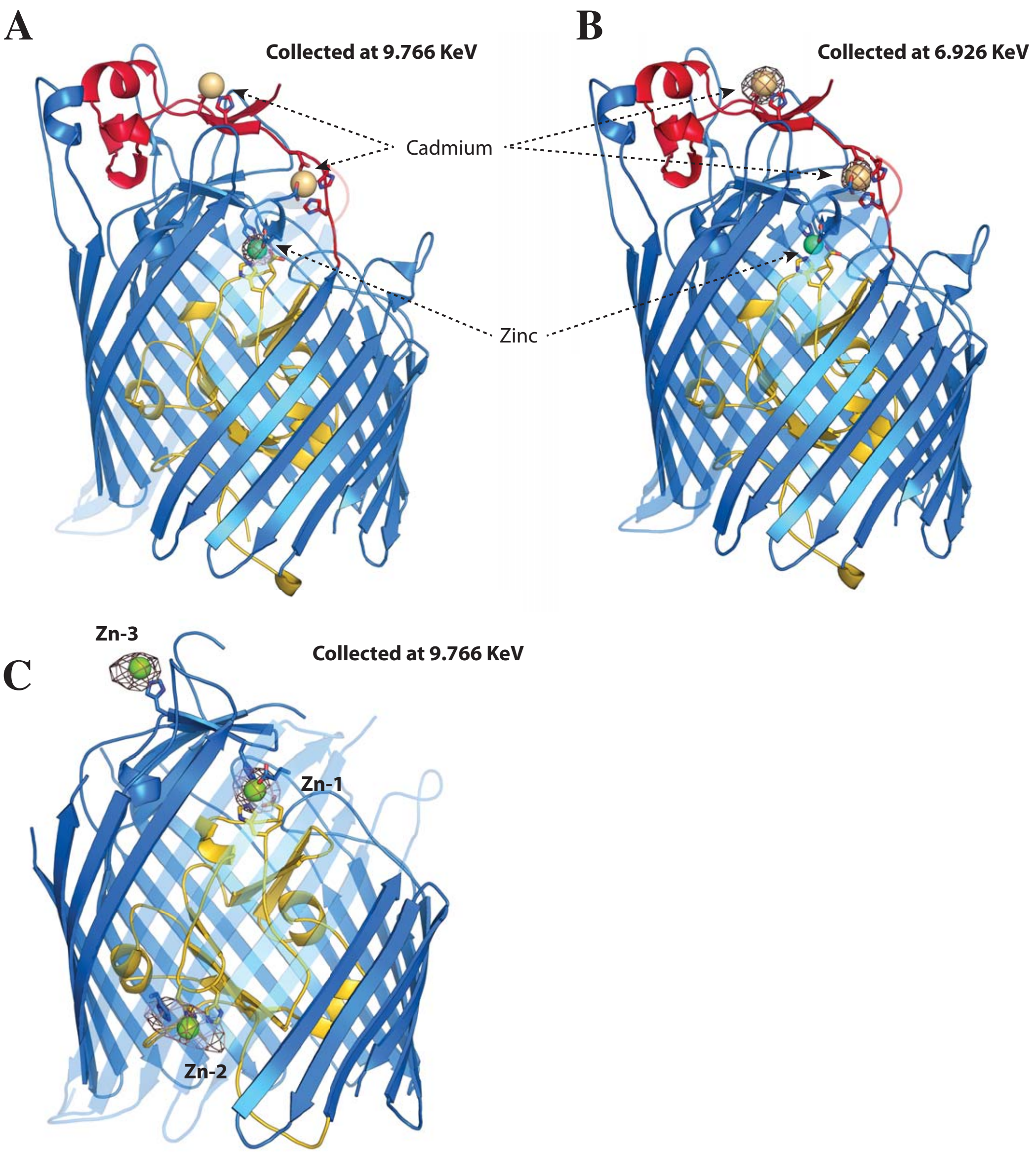

Supplementary figure 5: Anomalous difference map of Zn and Cd/Zn bound ZnuD. Anomalous difference map calculated from Cd/Zn-bound ZnuD crystals collected at 9.766 KeV (**A**) (excitation of zinc – map contoured at 14 sigma) and 6.926 KeV (**B**) (excitation of cadmium – map contoured at 7 sigma) confirm the identity of the cadmium and zinc ions. (**C**) The anomalous difference map calculated from a Zn-soaked ZnuD crystal collected at 9.766 KeV (excitation of zinc) confirms the presence of the 3 zinc molecules bound at the peripheral and periplasmic sides.

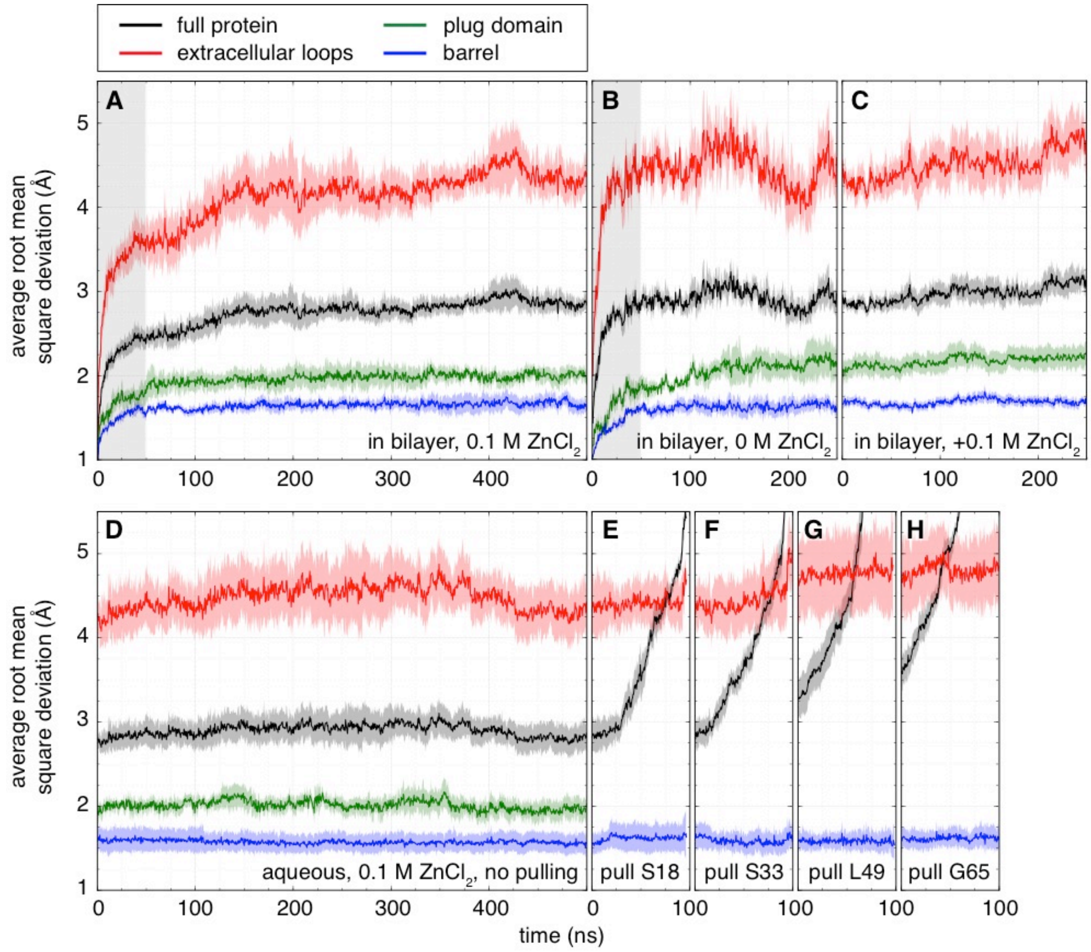

Supplementary figure 6: Average root-mean-square deviations (RMSD) of ZnuD from molecular simulations. The RMSD of full protein (black), extracellular loops (red), barrel (blue), and plug domain (green) relative to the pre-equilibrated simulation structure are shown as a function of simulation time. Shaded regions indicate the initial 50 ns of simulation data excluded from ensemble averages. Panels (**A**) and (**B**) present the RMSD of protein domains from the simulations with and without 100 mM $ZnCl_2$, respectively. Panel (**C**) illustrates the addition of 100 mM $ZnCl_2$ from the end of simulation (**B**). Panel (**D**) presents the RMSD of protein domains with $ZnCl_2$ restrained within an aqueous solution. Panels (**E**), (**F**), (**G**), (**H**) present the RMSD from simulations where the amino-terminal residues (S18, S33, L49 and G65) were unfolded from the plug domain by pulling in the periplasmic direction. These simulations were performed in succession starting from the end of the previous simulation (**D**). The sharp increase in RMSD of the full protein during pulling simulations is due to forced unfolding of the plug domain, which is omitted from panels (**E-H**).

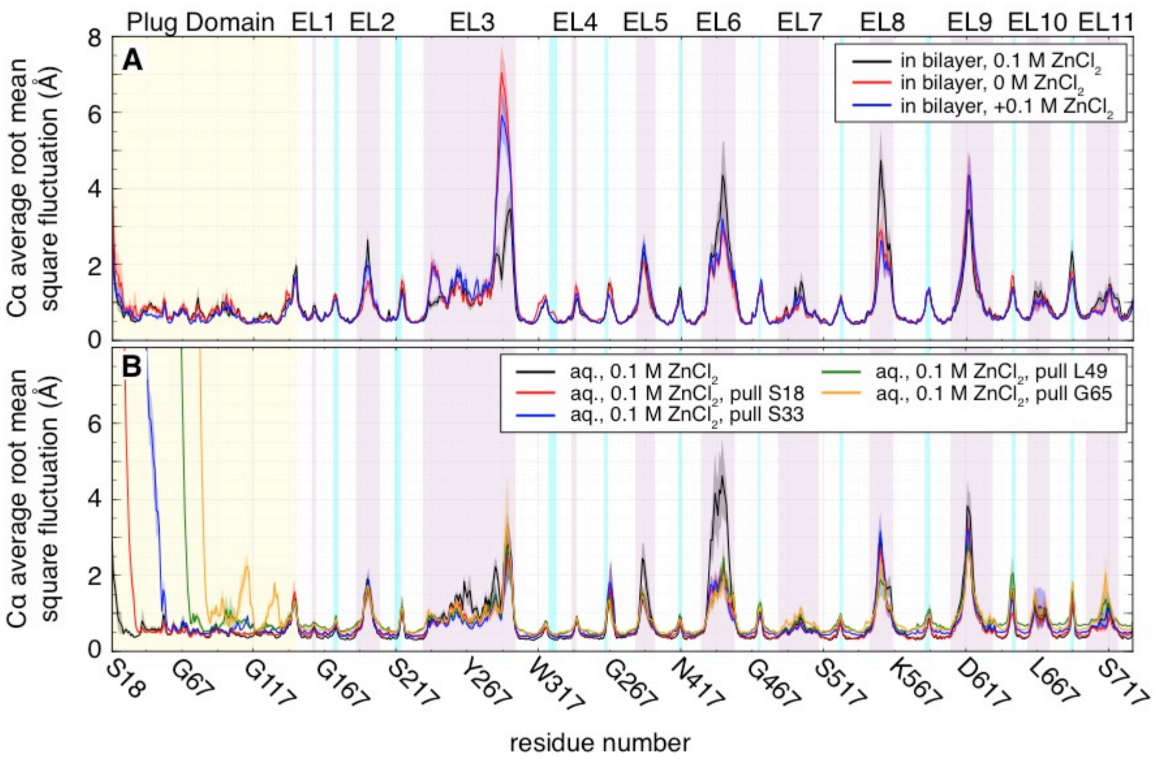

Supplementary figure 7: Average root-mean-square fluctuations (RMSF) of ZnuD. Average fluctuations are computed per residue after performing a β-barrel alignment to the pre-equilibrated simulation structure. Extracellular loops (shaded in purple), intracellular loops (shaded in aqua) and the plug domain (shaded in yellow) are shown based on secondary structure assignment of the ZnuD crystal structure. Panel (**A**) indicates the protein fluctuations per residue in bilayer simulations with and without $ZnCl_2$. Panel (**B**) indicates protein fluctuations per residue in aqueous solution with restrained beta barrel Cα atoms with and without unfolding of the plug domain.

Supplementary table S1: Summary of molecular dynamics simulations of ZnuD.

Num.	Simulation Name	Environment	Atoms (thousand)	Simulation Length (ns)	Equilibration Time (ns)	Simulation Repeats (#)
1	ZnuD + 0.1 M $ZnCl_2$	bilayer	~168	500	50	5
2	ZnuD + 0 M $ZnCl_2$	bilayer	~168	250	50	5
3	ZnuD + 0.1 M $ZnCl_2$ extended from series 2 after addition of salt	bilayer	~168	250	0	10
4	ZnuD + 0.1 M $ZnCl_2$	aqueous	~110	500	0	4
5	ZnuD + 0.1 M $ZnCl_2$ pulling S18 ~50 Å	aqueous	~110	~100	0	4
6	ZnuD + 0.1 M $ZnCl_2$ pulling S33 ~50 Å	aqueous	~110	~100	0	4
7	ZnuD + 0.1 M $ZnCl_2$ pulling L49 ~50 Å	aqueous	~110	~100	0	4
8	ZnuD + 0.1 M $ZnCl_2$ pulling G65 ~50 Å	aqueous	~110	~100	0	4

*Total simulation time ~9.85 microseconds.
